# FAERS database reveals latent signals of neuro-ophthalmic toxicity associated with immune checkpoint inhibitors: feature mining and clinical warnings

**DOI:** 10.3389/fimmu.2026.1696617

**Published:** 2026-07-21

**Authors:** Jiewen Li, Wen Zhang, Jing Lin, Lingjun Kong, Jun Wang, Wenqi Liu, Chunzhi Li, Dongna Zou

**Affiliations:** 1 Department of Pharmacy, Linyi People’s Hospital, Linyi, China; 2 Department of Pharmacy, Shandong Provincial Hospital Affiliated to Shandong First Medical University, Jinan, China; 3 Department of Pharmacy, Jinan Shizhong People’s Hospital, Jinan, China

**Keywords:** disproportionality analysis, FAERS, immune checkpoint inhibitors, neuro-ophthalmic toxicity, post-marketing drug safety monitoring

## Abstract

**Objective:**

To perform signal mining of neuro-ophthalmic immune-related adverse events (irAEs) associated with immune checkpoint inhibitors (ICIs) using the FDA Adverse Event Reporting System (FAERS), and to synthesize their clinical manifestations and temporal patterns, thereby providing evidence-based insights for optimizing clinical drug safety.

**Methods:**

Reports of neuro-ophthalmic irAEs associated with ICIs were extracted from the FAERS (Q1 2011 to Q4 2025). Signal detection was performed using disproportionality analysis methodologies, specifically the Reporting Odds Ratio (ROR) and Information Component (IC) algorithms, with predefined statistical thresholds for signal significance.

**Results:**

A total of 521 reports identified neuro-ophthalmic irAEs with ICIs as primary suspect drugs, predominantly affecting males and elderly patients At the High-Level Group Term (HLGT) level, positive signals were detected for pembrolizumab and avelumab. Preferred Term (PT) analysis revealed six significant signals, with eyelid ptosis being the most frequently reported event. The median time to onset was 28–38 days, with acute presentations (≤24 hours post-infusion) observed for pembrolizumab and nivolumab; both agents exhibited elevated case fatality proportions, with a higher proportion of male patients among fatal cases.

**Conclusion:**

This study delineated potential neuro-ophthalmic positive disproportionality signals across seven ICIs, revealing demographic variations in signal reporting frequency with higher susceptibility among males and elderly patients. Pembrolizumab and avelumab were associated with higher reporting frequencies of neuro-ophthalmic adverse events, which were related to poor visual outcomes. Consequently, prompt recognition and management of neuro-ophthalmic irAEs are imperative to enhance ICI safety profiles in clinical practice.

## 1 Introduction

Despite being a major breakthrough in cancer therapy, the clinical application of immune checkpoint inhibitors (ICIs) is limited by immune-related adverse events (irAEs), which occur in 54% - 76% of patients, are severe (Grade 3 - 4) in up to 28.6% of cases, and exhibit an overall incidence of up to 85.6% with monotherapy (1). Studies indicate that irAEs can affect multiple organs, such as the skin, endocrine glands, gastrointestinal tract, liver, and lungs, with marked interindividual heterogeneity in clinical manifestations (2–4). The incidence of ocular irAEs ranges from 2.8% to 4.3%. Neuro-ophthalmic irAEs are notably rarer and often omitted from drug prescribing information; however, they may induce afferent or efferent visual dysfunction. Importantly, efferent system irAEs (e.g., myasthenia gravis) exhibit a higher case fatality rate compared to afferent system irAEs (5). Therefore, intensive monitoring of adverse events and prompt intervention are imperative to ensure the safety and efficacy of ICI therapy, optimize patient outcomes, and sustain quality of life.

The immunosuppressive nature of the tumor microenvironment (TME) serves as a cornerstone mechanism enabling tumors to evade immune surveillance and facilitate progression. Within the TME, heightened expression of immune checkpoints such as cytotoxic T-lymphocyte-associated protein-4 (CTLA-4), programmed death-1 (PD-1), and programmed death-ligand 1 (PD-L1) disrupts the presentation of tumor antigens to cytotoxic T lymphocytes (CTLs), thereby inhibiting their immune surveillance capacity and ultimately permitting tumor immune escape (6). By blocking the overexpression of these immune checkpoints, ICIs neutralize immunosuppression and restore T-cell-mediated tumor recognition and cytotoxicity. However, this immune reactivation may trigger systemic hyperactivation, leading to organ-specific or systemic toxicities that manifest as irAEs (6, 7). The precise mechanisms triggering irAEs remain incompletely elucidated. Current evidence suggests associations with diminished peripheral immune tolerance and induction of organ-specific inflammatory processes (3, 8–10), potentially correlating with enhanced antitumor responses (9). Although preliminary observational data exist, large-scale clinical cohorts are necessary to definitively characterize the etiological drivers of irAEs.

The eye maintains unique immune privilege through multifaceted mechanisms—including physical barriers, localized immunosuppressive microenvironments, antigen sequestration, and limited lymphatic drainage (11, 12) — accounting for the low reporting frequency of ocular irAEs. However, ICIs may disrupt this equilibrium via target cross-reactivity, induction of autoantibodies, or inflammatory cell infiltration, thereby breaking immune tolerance and triggering aberrant immune responses against neural and ocular tissues (3, 4, 10, 13). Due to the non-regenerative nature of optic nerve fibers, failure to control immune-mediated inflammation may precipitate irreversible axonal degeneration within weeks, culminating in permanent vision loss (4). However, the biological mechanisms underlying neuro-ophthalmic toxicity induced by different types of ICIs remain unclear, and studies on the associated clinical characteristics and risk signals are still limited. Existing studies are mostly case reports or small-scale observations, lacking systematic evaluation of neuro-ophthalmic irAEs using large-scale real-world data.

Food and Drug Administration Adverse Event Reporting System (FAERS) is a leading global spontaneous reporting database that plays a critical role in monitoring the safety of marketed drugs and biological products. By employing advanced data mining algorithms, it enables quantitative assessment of potential associations between drugs and adverse events, supporting pharmacoepidemiological research and pharmacovigilance analyses. To date, no study has systematically investigated neuro-ophthalmic irAEs using the FAERS database. To address this gap, the present study intends to analyze real-world data from the FAERS database to conduct signal mining of potential associations between ICIs and neuro-ophthalmic irAEs, evaluate the correlation between different ICIs and the risk of these adverse reactions, and systematically describe clinical characteristics including patient baseline information, reported outcomes, and time-to-onset. This study aims to further elucidate the specific profiles and associated features of ICI-induced neuro-ophthalmic irAEs, thereby providing evidence-based references for clinical diagnosis and treatment decision-making.

## 2 Methods

### 2.1 Data source

The FAERS database is a publicly accessible spontaneous reporting system that compiles safety reports on marketed drugs and biologics from countries worldwide. These reports are submitted by healthcare professionals, drug manufacturers, patients, and other stakeholders such as attorneys. Since 2004, the FAERS database has been publicly available and updated quarterly. The database comprises seven key data files: demographic and administrative information (DEMO), drug information (DRUG), adverse events (REAC), patient outcomes (OUTC), report sources (RPSR), drug therapy starts and end dates (THER), and drug indications (INDI). The data for this study were sourced from the US FDA Adverse Event Reporting System (FAERS). The investigated target drugs were ICIs approved for marketing in the United States, with generic and brand names verified through the FDA Drug Approval Packages (https://www.accessdata.fda.gov/scripts/cder/daf) and DrugBank (https://go.drugbank.com). The ICIs of interest were CTLA-4 inhibitors (Ipilimumab), PD-1 inhibitors (Pembrolizumab, Nivolumab and Camrelizumab) and PD-L1 inhibitors (Atezolizumab, Avelumab and Durvalumab) (**Table 1**). Data were retrieved from Q1 2011 (corresponding to the earliest US ICI approval date) to Q4 2025. Following FDA-recommended deduplication protocols (14), reports were cleaned by (1): retaining entries with the latest FDA_DT (FDA receipt date) for identical CASEID values, and (2) selecting reports with the highest PRIMARYID when CASEID and FDA_DT were identical. Subsequently, the dataset was refined to include only reports where target ICIs were designated as Primary Suspect (PS) drugs.

**Table 1:** ICIs (US-approved) and approval dates.

Target	Generic name	Brand name	Approval year
CTLA-4	Ipilimumab	Yervoy	2011
PD-1	Pembrolizumab	Keytruda	2014
	Nivolumab	Opdivo	2014
	Cemiplimab	Libtayo	2018
PD-L1	Atezolizumab	Tecentriq	2016
	Avelumab	Bavencio	2017
	Durvalumab	Imfinzi	2017

### 2.2 Identification of relevant reports

All adverse events were coded using Preferred Terms (PTs) from the Medical Dictionary for Regulatory Activities (MedDRA, version 26.1), which allows standardized and precise coding of clinical conditions (15). Each PT is hierarchically categorized into High−Level Terms (HLT), High−Level Group Terms (HLGT) and corresponding System Organ Class (SOC) (16). Standardised MedDRA Queries (SMQs), an embedded MedDRA tool comprising grouped PTs describing clinically similar medical conditions, enable efficient retrieval of relevant adverse event reports. To ensure the specificity and accuracy of case inclusion, neuro−ophthalmic adverse events were extracted based on the HLGT “ Ocular neuromuscular disorders “ (Code: 10030061) within MedDRA version 26.1, and the corresponding specific PTs were validated via clinical expert consultation (**Table 2**).

**Table 2:** PTs contained in the narrow-scope search of”hyponatremia (SMQ)”.

Preferred terms	MedDRA code
Anisocoria	10002535
Argyll-Robertson pupils	10003093
Bell’s phenomenon	10071641
Binocular eye movement disorder	10061010
Blepharospasm	10005159
Ciliary muscle spasm	10009185
Cycloplegia	10011719
Diabetic ophthalmoplegia	10067567
Excessive eye blinking	10065166
Excessive ocular convergence	10053693
Extraocular muscle disorder	10053635
Eye movement disorder	10061129
Eye muscle entrapment	10065088
Eyelid function disorder	10061145
Eyelid myoclonus	10076060
Eyelid myokymia	10078745
Eyelid ptosis	10015995
Foster-Kennedy Syndrome	10017065
Gaze palsy	10056696
Heterophoria	10020015
Hippus	10057410
Holmes-Adie pupil	10020352
Iridoplegia	10067271
Lid lag	10024443
Miosis	10027646
Mydriasis	10028521
Ocular dysmetria	10063534
Ocular myasthenia	10049168
Oculogyric crisis	10030071
Ophthalmoplegia	10030875
Orbital apex syndrome	10066383
Oval pupil	10085734
Paralytic lagophthalmos	10033842
Parinaud syndrome	10072042
Pseudo-blepharoptosis	10037112
Pupil fixed	10037515
Pupillary deformity	10057426
Pupillary disorder	10037521
Pupillary reflex impaired	10037532
Pupillotonia	10050003
Strabismus	10042159
Superior oblique myokymia	10085542
Tolosa-Hunt syndrome	10051526

### 2.3 Statistical analysis

Descriptive analyses were performed to characterize the clinical profiles of patients experiencing ICI-related neuro-ophthalmic irAEs, including patient demographics (sex, body weight and age), reporter type, and patient outcomes. Categorical variables of all reports were presented as counts and percentages, while continuous variables were expressed as median (interquartile range, IQR). Data quality control included the exclusion of implausible physiological values (ages>120 years, weights >400 kg). Fatal neuro-ophthalmic irAEs were defined as cases with a patient outcome of death, and all remaining outcomes were classified as nonfatal.

Two specific indices, the reporting odds ratio (ROR) and Bayesian confidence propagation neural Network (BCPNN) information component (IC), were conducted to evaluate the potential neuro-ophthalmic irAEs risk linked to ICI therapy, with all other drugs/events documented in the FAERS database serving as the comparator. As a frequentist measure, ROR requires the lower bound of the 95% CI to be >1 for a positive signal. As a Bayesian measure, IC requires the lower bound of the 95% CI to be >0. ROR alleviates bias associated with sparse case numbers to generate stable estimations, while BCPNN improves analytical robustness through multi-source data integration and cross−validation; combining the two complementary algorithms enhances signal reliability and lowers the risk of false - positive findings (17). The relevant calculation formulas and criteria of ROR and IC are provided in online **Tables 3**, **4**. Consistent with prior disproportionality analyses, a potential safety signal was considered statistically significant if three criteria were met: the lower bound of the 95% CI for the ROR (ROR_025_) exceeded 1, the lower bound of the 95% CI for the IC (IC_025_) was greater than 0, and there were at least 3 case reports of specific neuro-ophthalmic irAEs (14, 18). An increase in the lower limit of the 95% CI of ROR, referred to as ROR, signifies a more robust signal, suggesting a heightened statistical correlation between the target drug and the target adverse event (17, 19).

**Table 3:** Two-by-two contingency table for combinations of ICIs and AE.

Item	Target AE	Other AE	Total
Target drug	a	b	a+b
Other drug	c	d	c+d
Total	a+c	b+d	a+b+c+d

Equation a: the number of reports containing both the target drug and target adverse drug reaction; Equation b: the number of reports containing other adverse drug reactions of the target drug; Equation c: the number of reports containing the target adverse drug reaction of other drugs; Equation d: the number of reports containing other drugs and other adverse drug reactions.

**Table 4:** Signal calculation formulas and criteria.

Method	Calculation formulas	Thresholds
ROR	ROR=(a/b)/(c/d)	a≧̸3 and 95% CI (lower limit)>1 suggests that 1 positive signal was generated.
IC	IC=log2a(a+b+c+d)/((a+c)(a+b)) IC025=eln(IC)-1.96(1/a+1/b+1/c+1/d)^0.5	IC025>0 suggests generation of 1 positive signal

ROR, Reporting Odds Ratio; IC.Information Component; CI, Confidence Interval; IC, Information Component; 95% CI, 95% confidence interval; IC_025_, the lower limit of 95% CI of the IC.

Time to onset of neuro-ophthalmic irAEs was defined as the interval between the onset date of neuro-ophthalmic irAEs (EVENT_DT in the DEMO dataset) and the initiation date of ICIs (START_DT in the THER dataset) (18). After excluding reports with missing or implausible date information, the Kaplan–Meier method was adopted to plot the cumulative incidence of ICI-related neuro-ophthalmic irAEs.

**Figure 1:**
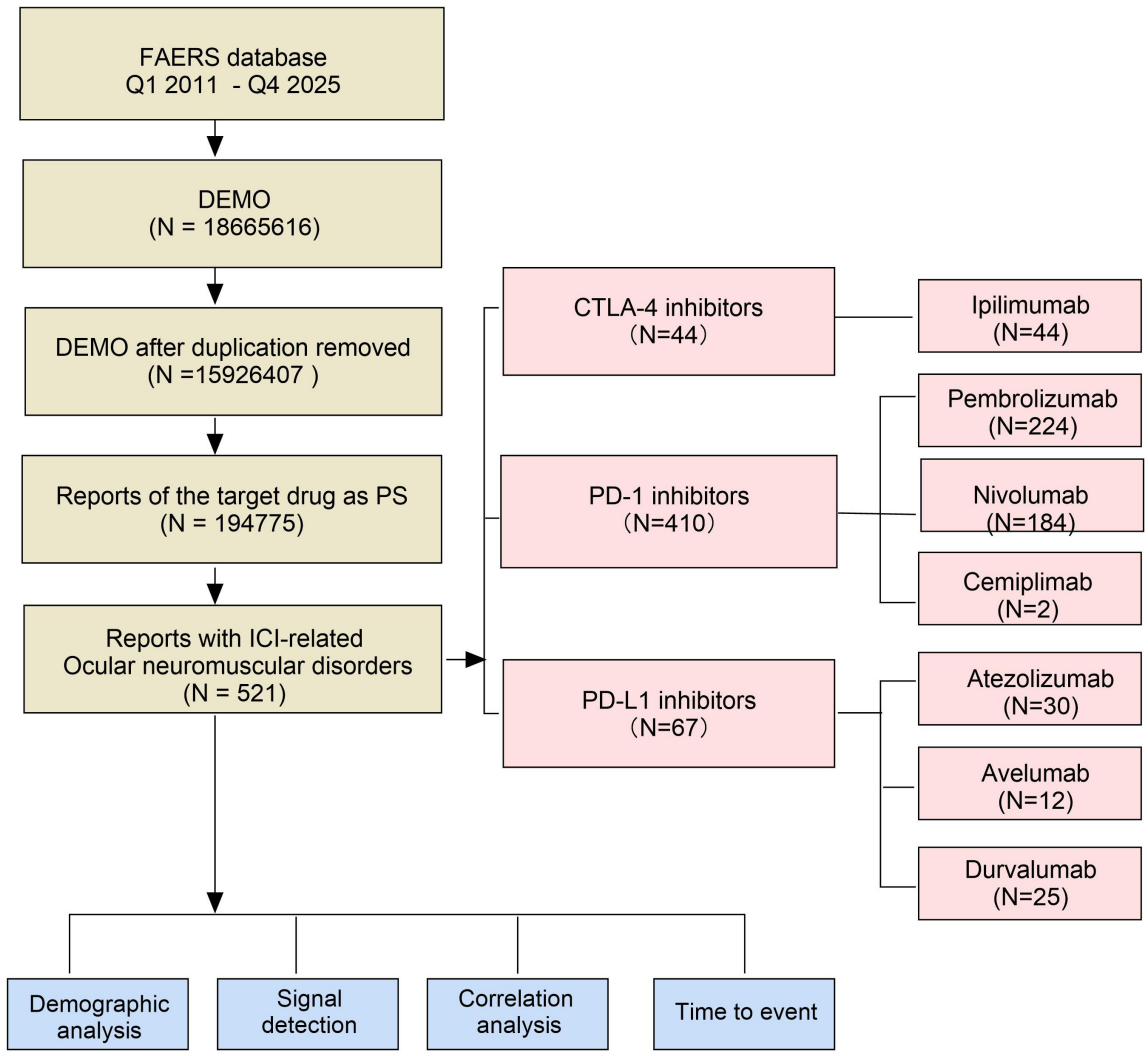
The flow chart of the study.

All data management, statistical analyses, and graphical representations in this study were performed using Microsoft Excel 2021, GraphPad Prism 10.1.2, and R software, version 4.3.3. A process diagram illustrating the study workflow is presentedin **Figure 1**.

## 3 Results

### 3.1 Key features of neuro-ophthalmic irAEs

A total of 521 reports of neuro-ophthalmic irAEs associated with ICIs were documented in the FAERS database from Q1 2011 to Q4 2025. PD-1 inhibitors accounted for 78.7% (410/521) of cases, followed by PD-L1 inhibitors at 12.9% (67/521), and CTLA-4 inhibitors at 8.4% (44/521). Seven specific agents were implicated: among PD-1 inhibitors, 224 cases (43.0%) were associated with pembrolizumab, 184 cases (35.3%) with nivolumab, and 2 cases (0.4%) with cemiplimab; among PD-L1 inhibitors, 30 cases (5.8%) were linked to atezolizumab, 12 cases (2.3%) to avelumab, and 25 cases (4.8%) to durvalumab; all CTLA-4 inhibitor cases (44 cases, 8.4%) were associated with ipilimumab. Healthcare professionals submitted the majority of reports (74.1%, 386/521), with physicians being the most frequent reporters (52.4%, 273/521). Detailed demographic characteristics of the cases involving neuro-ophthalmic irAEs are presented in **Table 5**.

**Table 5:** Basic characteristics of data reporting.

Characteristic		CTLA-4	PD-1			PD-L1			
		Ipilimumab (N = 44)	Pembrolizumab (N = 224)	Nivolumab (N = 184)	Cemiplimab(N = 2)	Atezolizumab (N = 30)	Avelumab(N = 12)	Durvalumab (N = 25)	Total (N = 521)
Sex	Female	19 (43.2%)	96 (42.9%)	67 (36.4%)	2 (100%)	10 (33.3%)	1 (8.3%)	12 (48.0%)	207(39.7%)
	Male	24 (54.5%)	123 (54.9%)	100 (54.3%)	0 (0%)	13 (43.3%)	11 (91.7%)	9 (36.0%)	280 (53.7%)
	Unknown	1 (2.3%)	5 (2.2%)	17 (9.2%)	0 (0%)	7 (23.3%)	0 (0%)	4 (16.0%)	34 (6.5%)
Weight	<50 kg	4 (9.1%)	6 (2.7%)	8 (4.3%)	0 (0%)	3 (10.0%)	0 (0%)	1 (4.0%)	22 (4.2%)
	>100 kg	1 (2.3%)	4 (1.8%)	5 (2.7%)	0 (0%)	1 (3.3%)	0 (0%)	1 (4.0%)	12 (2.3%)
	50~100 kg	10 (22.7%)	58 (25.9%)	70 (38.0%)	1 (50.0%)	15 (50.0%)	4 (33.3%)	9 (36.0%)	167 (32.1%)
	Unknown	29 (65.9%)	156 (69.6%)	101 (54.9%)	1 (50.0%)	11 (36.7%)	8 (66.7%)	14 (56.0%)	320 (61.4%)
Age	<18years	0 (0%)	0 (0%)	1 (0.5%)	0 (0%)	0 (0%)	0 (0%)	0 (0%)	1 (0.2%)
	>85years	1 (2.3%)	3 (1.3%)	5 (2.7%)	0 (0%)	0 (0%)	1 (8.3%)	0 (0%)	10 (1.9%)
	18~64.9years	14 (31.8%)	47 (21.0%)	50 (27.2%)	0 (0%)	7 (23.3%)	0 (0%)	5 (20.0%)	123 (23.6%)
	65~85years	25 (56.8%)	144 (64.3%)	99 (53.8%)	2 (100%)	17 (56.7%)	9 (75.0%)	12 (48.0%)	308 (59.1%)
	Unknown	4 (9.1%)	30 (13.4%)	29 (15.8%)	0 (0%)	6 (20.0%)	2 (16.7%)	8 (32.0%)	79 (15.2%)
Type of reporter	Physician	24 (54.5%)	101 (45.1%)	97 (52.7%)	1 (50.0%)	24 (80.0%)	11 (91.7%)	15 (60.0%)	273 (52.4%)
	Pharmacist	3 (6.8%)	16 (7.1%)	28 (15.2%)	0 (0%)	1 (3.3%)	0 (0%)	1 (4.0%)	49 (9.4%)
	Other health-professiona	3 (6.8%)	41 (18.3%)	15 (8.2%)	0 (0%)	3 (10.0%)	0 (0%)	2 (8.0%)	64 (12.3%)
	Consumer	8 (18.2%)	53 (23.7%)	25 (13.6%)	1 (50.0%)	1 (3.3%)	0 (0%)	1 (4.0%)	89 (17.1%)
	Missing	6 (13.6%)	13 (5.8%)	19 (10.3%)	0 (0%)	1 (3.3%)	1 (8.3%)	6 (24.0%)	46 (8.8%)
Outcome	Death	4 (9.1%)	51 (22.8%)	37 (20.1%)	1 (50.0%)	5 (16.7%)	1 (8.3%)	3 (12.0%)	102 (19.6.%)
	Disability	0 (0%)	2 (0.9%)	1 (0.5%)	0 (0%)	0 (0%)	1 (8.3%)	0 (0%)	4 (0.8%)
	Hospitalization	19 (43.2%)	88 (39.3%)	83 (45.1%)	1 (50.0%)	14 (46.7%)	4 (33.3%)	12 (48.0%)	221 (42.4%)
	Life-Threatening	4 (9.1%)	18 (8.0%)	17 (9.2%)	0 (0%)	3 (10.0%)	0 (0%)	2 (8.0%)	44 (8.4%)
	Other	17 (38.6%)	65 (29.0%)	46 (25.0%)	0 (0%)	8 (26.7%)	6 (50.0%)	8 (32.0%)	150 (28.8%)

The same patient may experience multiple ADEs.

### 3.2 Clinical characteristics of neuro-ophthalmic irAEs

Analysis of patient characteristics from neuro-ophthalmic irAE reports (**Table 5**) revealed a higher proportion of males (53.7%, 280/521) compared to females (39.7%, 207/521). The majority of cases (59.1%, 308/521) occurred in patients aged ≥ 65 years, while only 1 case was documented in the pediatric/adolescent population, involving a patient receiving pembrolizumab. Hospitalization was the most frequent outcome (42.4%, 221/521). Serious outcomes (death, disability, or life-threatening events) accounted for 28.8% (150/521) of reports. Death events were reported for all ICIs. Notably, none of the reports included documentation of recovery or resolution from neuro-ophthalmic irAEs.

### 3.3 Detection of signals

Disproportionality analysis using ROR and IC methods revealed statistically significant disproportional associations between ICIs and neuro-ophthalmic irAEs at the HLGT level. Pembrolizumab (40.5%, 318/785) and nivolumab (39.6%, 311/785) exhibited the highest report frequencies. Crucially, only pembrolizumab (ROR = 1.45 (1.3 - 1.62), IC = 0.53 (0.37)) and avelumab (ROR = 2.12 (1.23 - 3.65), IC = 1.08 (0.2)) generated positive signals (**Table 6**). Notably, avelumab had a small sample size with a relatively wide confidence interval, which may cause unstable signal estimation. Therefore, its positive statistical association should be interpreted cautiously and does not necessarily indicate a higher actual clinical risk.

**Table 6:** Results of correlation analysis between different ICIs and neuro-ophthalmic toxicity.

Drugs	Number	ROR(95%CI)	IC(IC025)	whether a signal is generated
Ipilimumab	68	1.06 (0.84 - 1.35)	0.08 (-0.26)	No
Pembrolizumab	318	1.45 (1.3 - 1.62)	0.53 (0.37)	Yes
Nivolumab	311	1.09 (0.97 - 1.21)	0.12 (-0.04)	No
Cemiplimab	2	0.66 (0.16 - 2.62)	-0.61 (-2.1)	No
Atezolizumab	42	0.54 (0.4 - 0.74)	-0.87 (-1.3)	No
Avelumab	13	2.12 (1.23 - 3.65)	1.08 (0.2)	Yes
Durvalumab	31	0.81 (0.57 - 1.15)	-0.31 (-0.81)	No

**Figure 2:**
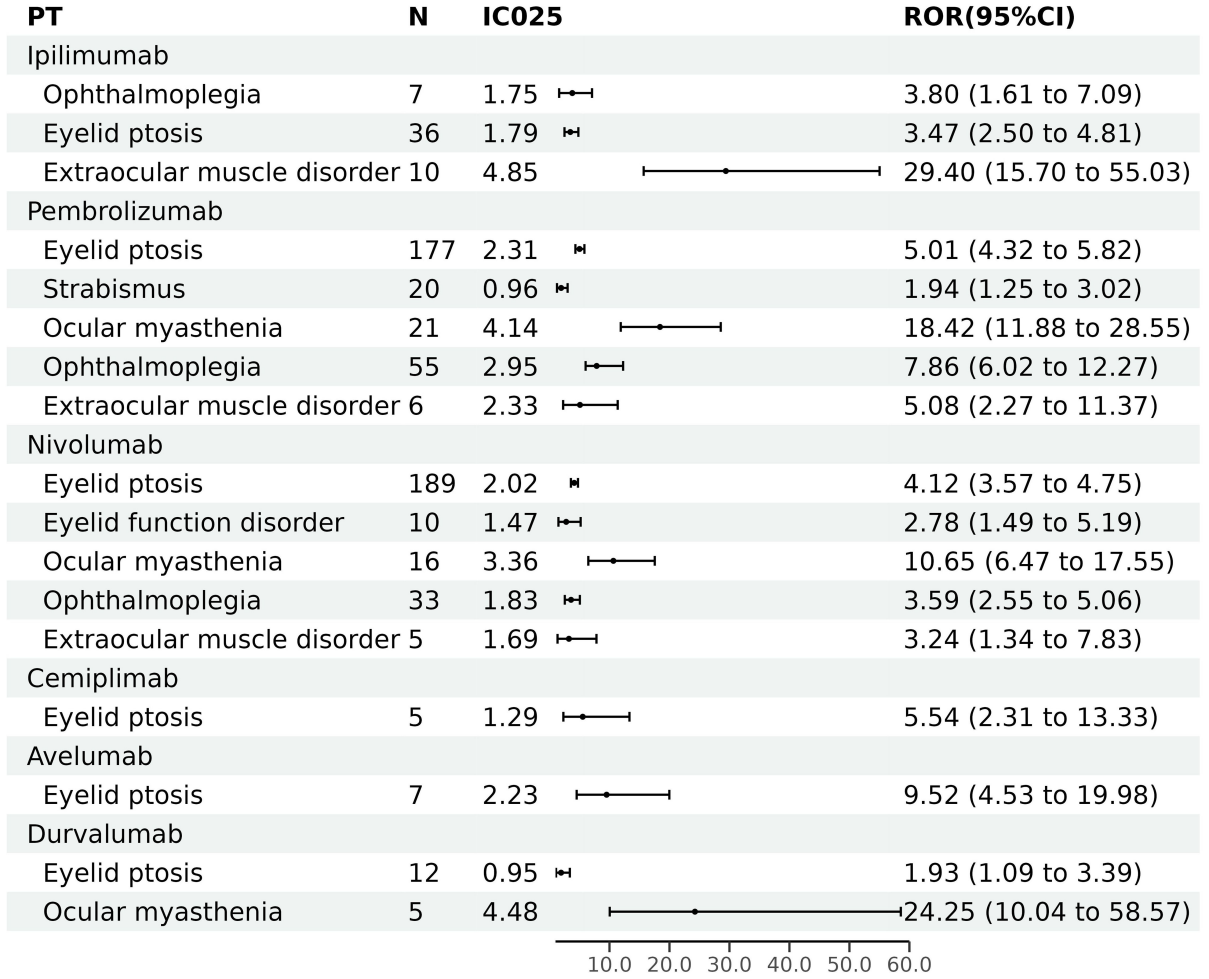
Results of correlation analysis of neuro-ophthalmic irAEs at the PT level.

Disproportionality analysis at the PT level under the HLGT identified six positive signals: eyelid ptosis, extraocular muscle disorder, strabismus, ocular myasthenia, ophthalmoplegia, and eyelid function disorder. As atezolizumab-associated neuro-ophthalmic irAEs yielded fewer than three reports meeting positive signal criteria, it was excluded from this analysis, resulting in six ICIs being included, with the results presented in **Figure 2**. Both pembrolizumab and nivolumab generated five signals each, sharing four common PTs: eyelid ptosis, ocular myasthenia, ophthalmoplegia, and extraocular muscle disorder. Eyelid ptosis was the most frequently reported event (426/614, 69.4%), involving all six ICIs, with nivolumab (30.8%, 189/614) and pembrolizumab (28.8%, 177/614) constituting the majority of cases. Pembrolizumab demonstrated the strongest ptosis signal (ROR = 5.01 (4.32 - 5.82), IC = 2.31). Ocular myasthenia signals were robust across three ICIs (pembrolizumab, nivolumab, durvalumab), with durvalumab exhibiting the highest signal strength (ROR = 24.25 (10.04 - 58.57), IC = 4.58). Of note, several ICIs, including cemiplimab and avelumab, were represented by very small sample sizes, resulting in unstable signal estimates and wide confidence intervals for their corresponding adverse event signals. Therefore, these positive statistical signals should be interpreted with caution and do not definitively indicate elevated clinical toxicity risk. The limited number of cases for certain signals may introduce uncertainty, warranting further validation studies.

### 3.4 Time to onset of neuro-ophthalmic irAEs

**Figure 3:**
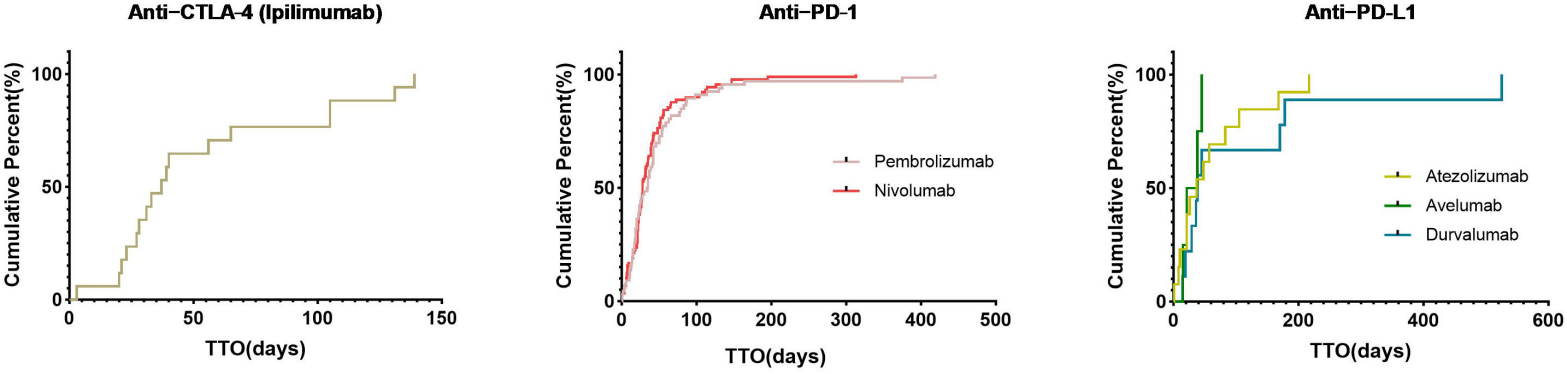
Number of reports and onset time of neuro-ophthalmic irAEs associated with different types of ICIs

A total of 204 reports with clearly documented time-to-onset data for neuro-ophthalmic irAEs were included in this analysis, accounting for 39.2% (204/521) of all reports. The median time to onset ranged from 28–38 days across ICIs, with most events clustering around 5 weeks post-initiation (**Table 7**). It is noteworthy that certain agents, including pembrolizumab, nivolumab, and atezolizumab, were associated with neuro-ophthalmic irAEs occurring immediately after treatment initiation. In contrast, avelumab and durvalumab demonstrated a delayed onset pattern for these adverse events. Additionally, pembrolizumab and nivolumab were also linked to cases with extended time-to-onset. The cumulative reporting frequency rates of various neuro-ophthalmic irAEs are shown in **Figure 3**. Given that complete onset data were available for merely 39.2% of all cases, the reliability of temporal analysis and conclusions regarding acute versus delayed onset patterns is limited, and thus the summarized onset characteristics above should be interpreted prudently owing to insufficient temporal information.

**Table 7:** Number of reports and time to onset of different neuro-ophthalmic irAEs.

Time to Onset		CTLA-4	PD-1			PD-L1		
		Ipilimumab (N = 17)	Pembrolizumab (N = 68)	Nivolumab (N = 92)	Cemiplimab (N = 1)	Atezolizumab (N = 13)	Avelumab (N = 4)	Durvalumab (N = 9)
Group of TTO	0–30 days	6	34	50		6	2	3
	31–60 days	6	20	28	1	3	2	3
	61–90 days	1	7	5		1		
	91–120 days	2	2	4		1		
	121–150 days	2	2	3				
	151–180 days		1			1		2
	181–360 days					1		
	>360 days		2	2				1
Median TTO		37 (3 -139)	34.5 (1-419)	28 (1-313)	59	37 (1-217)	29.5 (15-45)	38 (14-525)

### 3.5 Outcome of neuro-ophthalmic irAEs

**Figure 4:**
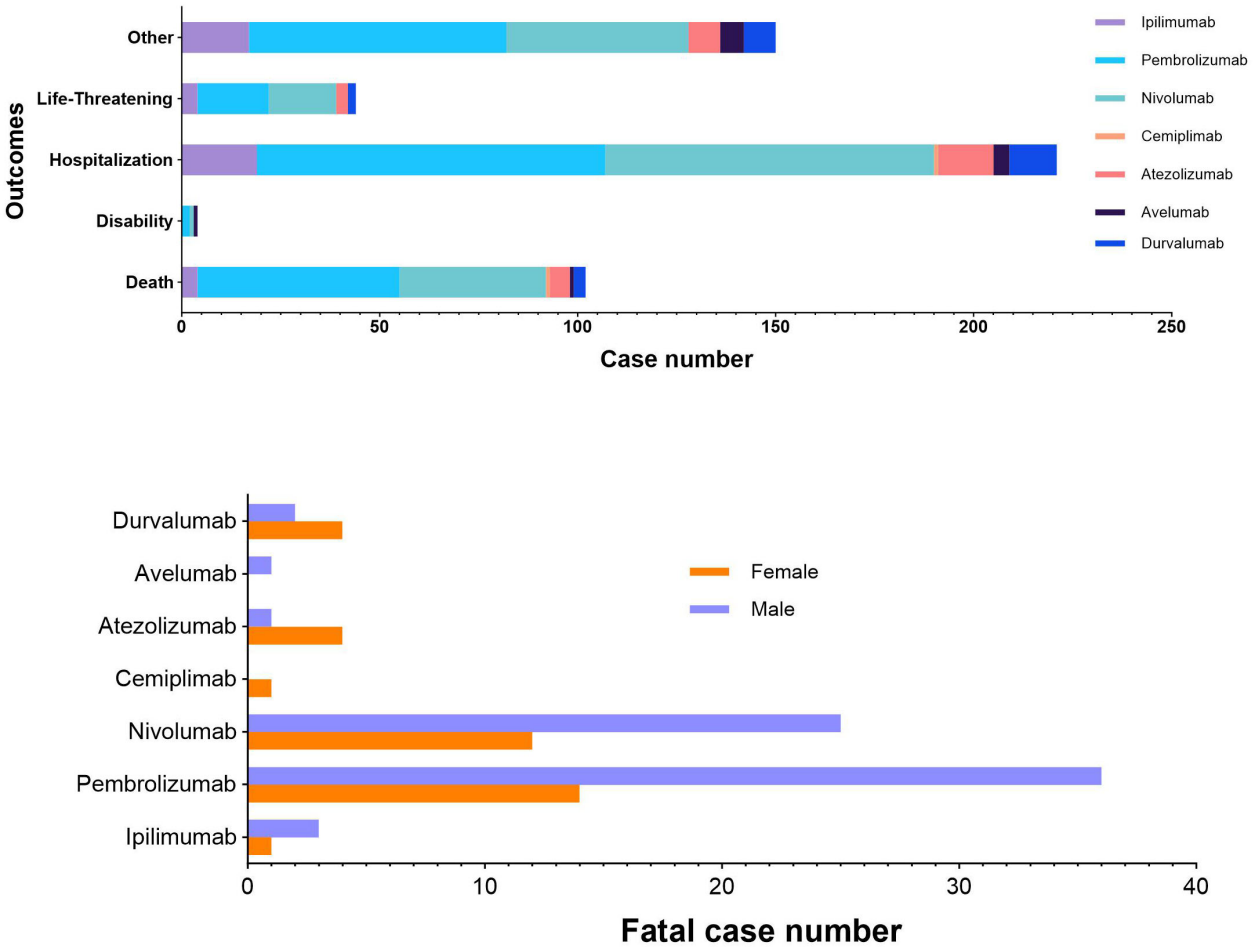
Outcomes of neuro-ophthalmic irAEs associated with different ICIs.

To further assess the severity of adverse events, this study analyzed cases of fatal or life-threatening neuro-ophthalmic irAEs associated with different ICI monotherapies. The results showed that pembrolizumab and nivolumab had the highest number of reported cases as well as the highest case fatality proportions, at 22.8% (51/224) and 20.1% (37/184), respectively. The case fatality proportions for other ICIs ranged from 8.3% to 19.6%. The proportions of patients with neuro-ophthalmic irAE-related serious outcomes (death, disability, or life-threatening events) are listed in descending order as follows: Atezolizumab (26.7%, 8/30) > pembrolizumab (23.7%, 55/224) > nivolumab (20.7%, 38/184) > durvalumab (20.0%, 5/25) > ipilimumab (18.2%, 8/44) > avelumab (16.7%, 2/12). Notably, as shown in **Figure 4**, among patients who died from neuro-ophthalmic irAEs related to CTLA-4 and PD-1 inhibitors, males accounted for a higher proportion (69.6%, 64/92) than females (30.4%, 28/92). By contrast, among those who died from neuro-ophthalmic irAEs associated with PD-L1 inhibitors, females accounted for a higher proportion (66.7%, 8/12) than males (33.3%, 4/12). Derived exclusively from reported cases, this indicator does not equal the true mortality risk and requires cautious interpretation. The small sample size also leads to uncertain results, which need to be verified in future studies. See **Figure 4** for details.

## 4 Discussion

With growing public awareness of adverse reactions associated with drug therapies, Eye-irAEs of ophthalmic and neuro-ophthalmic have become a noteworthy safety concern for both patients and healthcare providers (20).To address the limitations of clinical trials and case reports in investigating ICI-induced neuro-ophthalmic irAEs, our study is the first to leverage the FAERS database and apply multiple disproportionality analysis methods including ROR and BCPNN to quantify the association between ICIs and neuro-ophthalmic toxicity. We systematically analyzed 521 reports related to neuro-ophthalmic toxicity, with six ICIs listed as the primary suspected drugs. Baseline characteristics of patients and reports were described, and differences in the onset time of neuro-ophthalmic irAEs associated with these inhibitors were explored. Additionally, the distribution of ICIs was examined at both HLGT and PT levels to provide a more detailed safety profile.

Neuro-ophthalmic disorders represent relatively prevalent ocular In a pharmacovigilance analysis by Bomze et al., optic neuropathies were identified in 8.4% of ICI-treated patients, highlighting their clinical significance among immune-related visual complications (21). In a systematic review of 290 published cases, Martens et al. identified neurological abnormalities in 24.5% of patients with ocular irAEs, highlighting the frequent co-occurrence of neuro-ophthalmic involvement in ocular immune toxicity (22). This study identified 521 reports of neuro-ophthalmic irAEs, representing a significantly lower reporting frequency compared to other irAE categories. Critically, the absence of explicit labeling for neuro-ophthalmic toxicities in current drug prescribing information may impede recognition across diverse populations, potentially exacerbating risks of irreversible axonal degeneration and permanent vision loss. Physician-driven reporting predominated, underscoring the necessity for specialized pharmacovigilance frameworks to mitigate diagnostic delays and therapeutic challenges. In our cohort, neuro-ophthalmic irAEs predominated in male patients (53.7% vs. 39.7% females), aligning with prior epidemiological reports (22, 23). This gender disparity may involve dual immunomodulatory effects of sex hormones on ocular immunity (24). Pala et al., utilizing the EU pharmacovigilance database for analysis of ICI-associated adverse events, demonstrated a higher proportion of male patients reporting adverse events following treatment with CTLA-4 or PD-1 inhibitors (25). Current evidence remains insufficient to establish gender as a determinant of irAE reporting frequency (26). Clinical vigilance is warranted for neuro-ophthalmic irAEs in male patients following ICI therapy. Advanced age is a well-established risk factor for multiple cancers and ophthalmic disorders (27, 28). This study further demonstrates that elderly populations exhibit higher reporting frequency of neuro-ophthalmic irAEs, necessitating enhanced monitoring for ICI-treated older adults with particular attention to clinical manifestations of neuro-ophthalmic pathologies post-therapy.

Positive disproportionality signals at the HLGT level were identified for pembrolizumab and avelumab but not for other ICIs, probably because reporters used inconsistent PT coding for neuro-ophthalmic irAEs. The diagnostic challenge stems from substantial clinical overlap between neuro-ophthalmic irAEs (e.g., optic neuritis, cranial neuropathies, ophthalmoplegia) and primary CNS disorders, complicating causality assessment (29). Analysis at the PT level identified six positive signals, spanning six distinct therapeutic agent, indicating that ICIs commonly used in clinical practice can induce neuro-ophthalmic irAEs. PD-1 inhibitors exhibited the highest signal diversity and frequency, followed by CTLA-4 inhibitors. A systematic review of 109 published monotherapy case reportson neuro-ophthalmic irAEs identified pembrolizumab as the most frequently implicated agent, followed by nivolumab and ipilimumab (30). These pharmacovigilance profiles align with our study findings, einforcing PD-1 inhibitors’ predominant association with neuro-ophthalmic toxicities. CTLA-4 inhibitors were the first immune checkpoint inhibitors approved for clinical use. Mechanistically, theyexert upstream immunomodulation by competitively inhibiting CD28-B7 co-stimulation during T-cell priming, contrasting with PD-1/PD-L1 inhibitors that primarily regulate effector-phase T-cell activity Consistent with this broader immunologic impact, CTLA-4 inhibitors demonstrate the highest reported reporting frequency of irAEs (31). This study found a higher reporting frequency of neuro-ophthalmic irAEs with PD-1 inhibitors than CTLA-4 inhibitors, while pembrolizumab demonstrated marginally stronger signal strength than nivolumab. At the PT level, the PD-L1 inhibitor avelumab was associated with a positive signal for ptosis, suggesting that drooping of the upper eyelid represents a major manifestation of its high neuro-ophthalmic toxicity. Avelumab not only activates adaptive immunity by blocking the PD-1/PD-L1 inhibitory pathway, but also enhances innate immune responses and improves immune cell function through Fc domain-mediated, Fc receptor-dependent mechanisms, thereby exerting antitumor effects (32). While this dual mechanism synergistically enhances antitumor immune responses, it may also disrupt self-tolerance via off-target cross-reactivity (3), potentially leading to neuro-ophthalmic irAEs. The precise mechanisms underlying these effects require further investigation. It is noteworthy that neuro-ophthalmic irAEs may arise within 24 hours post-infusion of PD-1/PD-L1 inhibitors such as pembrolizumab, nivolumab, and atezolizumab, whereas avelumab and durvalumab are associated with a delayed onset, typically occurring approximately 2 weeks post-dosing. Collectively, the pathogenesis of neuro-ophthalmic irAEs exhibits high agent-specificity, yet remains poorly characterized mechanistically. Consequently, heightened clinical vigilance is warranted when administering ICIs such as pembrolizumab, nivolumab, and avelumab.

Eyelid ptosis was the most frequently reported signal and involved the broadest spectrum of ICI classes, often correlating with multiple ophthalmic comorbidities and poor visual outcomes. Fernández et al. reported two cases of oculomotor nerve palsy in which eyelid ptosis persisted for 6 months post-glucocorticoid therapy, suggesting the occurrence of irreversible axonal damage (29). Pembrolizumab and nivolumab generated four identical positive signals: eyelid ptosis, ocular myasthenia, ophthalmoplegia, and extraocular muscle disorder. Eyelid ptosis and diplopia—which may manifest as ocular myasthenia or ophthalmoplegia—represent the most common clinical features of ocular myasthenia gravis. Prior studies indicate that ICI-associated myasthenia gravis occurs in approximately 0.47% of patients, predominantly with anti-PD-1 agents (notably nivolumab and pembrolizumab) (30), aligning with our findings. Myasthenia gravis is a life-threatening irAE with high mortality potential, characterized by progressive muscle weakness affecting ocular, bulbar, and limb muscles. It frequently coexists with myositis and/or myocarditis, may progress to respiratory failure, and exhibits early onset (median time: 29 days post-ICI initiation) (33, 34). Furthermore, extraocular myopathy frequently associates with orbital myositis, involving inflammatory damage to extraocular muscles themselves, which may coexist with or occur independently of ocular myasthenia gravi (35). Orbital myositis potentially correlates with life-threatening myocarditis and diaphragmitis (34, 36). This study additionally found that neuro-ophthalmic irAEs may manifest within 24 hours post-infusion of pembrolizumab, nivolumab, or atezolizumab. Therefore, screening protocols for high-risk patients should incorporate comprehensive ophthalmic examinations; identifying the six key symptoms detected in this study—including ptosis and diplopia—will substantially facilitate proactive surveillance of patients at risk of progressing to severe irAEs.

This study identified a median onset time of neuro-ophthalmic irAEs ranging from 28–38 days post-ICI initiation, with significant temporal variations across different ICIs. Notably, agents such as pembrolizumab, nivolumab, and atezolizumab demonstrated potential for immediate manifestation (≤24 hours post-infusion). For avelumab-associated neuro-ophthalmic irAEs, the median time to onset was 29.5 days post-initiation. Published literature indicates that its predominant irAEs involve systemic and nervous system disorders, frequently manifesting ≥2 months post-dosing (37). This indicates higher reporting frequencies of neuro-ophthalmic irAEs during the early phase of ICI therapy, particularly within the initial 5 weeks post-initiation. Consequently, enhanced monitoring for ocular adverse events is warranted, especially during the early treatment phase, to enable prompt intervention and mitigate progression to neuro-ophthalmic complications. Notably, pembrolizumab may induce neuro-ophthalmic irAEs after extended treatment durations, while both pembrolizumab and nivolumab demonstrate higher case fatality proportions in neuro-ophthalmic irAEs compared to other ICIs. This phenomenon may stem from delayed pharmacodynamic effects—where chronic immune-related adverse events (delayed irAEs) can manifest months after immunotherapy cessation (38). Delayed irAEs can involve multiple organ systems, including endocrine, rheumatological, gastrointestinal, dermatological, and neurological systems, with some persisting for over 18 months (31, 38, 39). Ocular delayed irAEs are relatively infrequently reported. A multicenter retrospective study of 387 high-risk patients with resected melanoma found that 4 patients developed delayed ocular irAEs following adjuvant anti-PD-1 therapy, primarily manifesting as uveitis (40). Studies demonstrate that a single dose of nivolumab maintains 80% PD-1 receptor occupancy on T-cells at 90 days post-administration (41). This indicates that although neuro-ophthalmic irAEs frequently occur during early treatment phases, clinical decision-making must rigorously account for delayed irAE risks, particularly in long-term survivors requiring extended surveillance and management​​.

In addition, we identified significant gender disparities among fatal cases across different ICI agents, primarily characterized as follows: Among patients who succumbed to neuro-ophthalmic irAEs following CTLA-4 or PD-1 inhibitor treatment, males accounted for a higher proportion (69.6%, 64/92) compared to females (30.4%, 28/92), which aligns with prior research findings (23, 30); in contrast, among patients who died of neuro-ophthalmic irAEs associated with PD-L1 inhibitors, females represented a larger share (66.7%, 8/12) relative to males (33.3%, 4/12). In existing case reports, fatal complications in male patients were more concentrated in myasthenia gravis, whereas female fatal cases were predominantly complicated by multisystem immune damage (myositis/myocarditis) (23, 30). Consequently, the clinical management of neuro-ophthalmic irAEs should incorporate considerations of clinical manifestations, administered ICI agents, gender, and underlying diseases. Upon the occurrence of neuro-ophthalmic irAEs, a multidisciplinary assessment involving oncologists and ophthalmologists is warranted, and temporary discontinuation of ICI treatment should be contemplated. The decision to resume ICI therapy ought to be reassessed once ocular symptoms are controlled. Symptomatic management is grounded in glucocorticoids: topical administration (eye drops, intravitreal injection) is preferred for mild cases, while systemic administration (intravenous methylprednisolone 1000 mg/day for 3–5 days or oral prednisone 1 mg/kg/day) is indicated for severe cases (20, 23). For patients refractory to glucocorticoids, combination therapy with intravenous immunoglobulin or plasmapheresis should be initiated (20).

When interpreting the present study, several limitations inherent to the FAERS database warrant particular attention. First, as a spontaneous reporting system, the FAERS database may introduce multiple biases, including underreporting, delayed reporting, duplicate entries, and inaccurate information. Even with large datasets and conventional statistical methods, handling missing values and outliers may propagate additional bias. Second, FAERS solely captures reported drug-adverse event associations; frequency-based disproportionality analyses cannot estimate true population reporting rate. Third, the FAERS database lacks data on patient drug exposure and detailed clinical information. Therefore, we were unable to adjust for major confounding factors including tumor type, treatment duration, line of therapy and combination medication regimens in this study. Furthermore, reports do not require causality verification between drugs and adverse events, and the absence of detailed patient baseline characteristics and treatment specifics poses challenges for analyzing complex clinical scenarios. Thus, findings must be interpreted cautiously, with limitations factored into conclusions. Future prospective studies should validate causal links between ICIs and neuro-ophthalmic irAEs, identify predictive biomarkers, and elucidate molecular mechanisms driving these toxicities. This research direction will contribute to optimizing clinical management strategies for adverse events and provide guidance for treatment selection.

## 5 Conclusion

In conclusion, this pharmacovigilance study analyzed the potential risk of neuro-ophthalmic toxicity associated with seven ICIs from Q1–2011 to Q4 2025.Neuro-ophthalmic irAEs demonstrated higher reporting frequency in males and elderly patients, with a median onset time of 28–38 days post-treatment. Pembrolizumab and avelumab showed prominent positive disproportionality signals for neuro-ophthalmic adverse events, with ptosis as the predominant manifestation, which may lead to poor visual prognosis. Consequently, clinicians should prioritize early recognition and prompt management of neuro-ophthalmic irAEs to enhance ICI safety profiles. However, limitations inherent to the FAERS spontaneous reporting system necessitate cautious interpretation, warranting validation through prospective studies.

## Data Availability

The datasets presented in this study can be found in online repositories. The names of the repository/repositories and accession number(s) can be found in the article/supplementary material.
